# Persistent Depletion of Neuroprotective Factors Accompanies Neuroinflammatory, Neurodegenerative, and Vascular Remodeling Spectra in Serum Three Months after Non-Emergent Cardiac Surgery

**DOI:** 10.3390/biomedicines10102364

**Published:** 2022-09-22

**Authors:** Krzysztof Laudanski, Da Liu, Tony Okeke, Mariana Restrepo, Wilson Y. Szeto

**Affiliations:** 1 Department of Anesthesiology and Critical Care, University of Pennsylvania, Philadelphia, PA 19104, USA; 2 Department of Neurology, University of Pennsylvania, Philadelphia, PA 19104, USA; 3 Leonard Davis Institute for Health Economics, University of Pennsylvania, Philadelphia, PA 19104, USA; 4 Department of Obstetrics and Gynecology, Shengjing Hospital of China Medical University, Shenyang 110055, China; 5 Department of Bioengineering, Drexel University, Philadelphia, PA 19104, USA; 6 College of Arts and Sciences, University of Pennsylvania, Philadelphia, PA 19104, USA; 7 Division of Cardiovascular Surgery, Department of Surgery, University of Pennsylvania, Philadelphia, PA 19104, USA

**Keywords:** cardiac surgery, neuroprotection, neurodegeneration, neuroinflammation, atypical neurodegeneration NF-L, GFAP, apoE, fetuin, clusterin, amyloid, NCAM-1, CCL28

## Abstract

We hypothesized that the persistent depletion of neuroprotective markers accompanies neuroinflammation and neurodegeneration in patients after cardiac surgery. A total of 158 patients underwent elective heart surgery with their blood collected before surgery (t_baseline_) and 24 h (t_24hr_), seven days (t_7d_), and three months (t_3m_) post-surgery. The patients’ serum was measured for markers of neurodegeneration (τau, τaup181–183, amyloid β1-40/β2-42, and S100), atypical neurodegeneration (KLK6 and NRGN), neuro-injury (neurofilament light/heavy, UC-HL, and GFAP), neuroinflammation (YKL-40 and TDP-43), peripheral nerve damage (NCAM-1), neuroprotection (apoE4, BDNF, fetuin, and clusterin), and vascular smoldering inflammation (C-reactive protein, CCL-28 IL-6, and IL-8). The mortality at 28 days, incidence of cerebrovascular accidents (CVA), and functional status were followed for three months. The levels of amyloid β1-40/β1-42 and NF-L were significantly elevated at all time points. The levels of τau, S100, KLK6, NRGN, and NCAM-1 were significantly elevated at 24 h. A cluster analysis demonstrated groupings around amyloids, KLK6, and NCAM-1. YKL-40, but not TDP-43, was significantly elevated across all time points. BDNF, apoE4, fetuin, and clusterin levels were significantly diminished long-term. IL-6 and IL-8 levles returned to baseline at t_3m_. The levels of CRP, CCL-28, and Hsp-70 remained elevated. At 3 months, 8.2% of the patients experienced a stroke, with transfusion volume being a significant variable. Cardiac-surgery patients exhibited persistent peripheral and neuronal inflammation, blood vessel remodeling, and the depletion of neuroprotective factors 3 months post-procedure.

## 1. Introduction

Over 340,000 patients undergo heart surgery annually in the United States [1,2]. The progress of their recovery is determined by comorbidities, age, and circumstances relating to surgery [3,4,5,6,7,8,9]. Cardiac surgery patients may also experience cognitive-related post-surgical effects due to surgery-associated inflammation, peri-operative hypoperfusion, and free radicals creating a neurotoxic environment as evidenced by the peri-operative release of neurofilament light (NF-L) and protein S100 [10,11,12,13]. Furthermore, the persistence of local post-surgical neuroinflammation compounds neuronal damage and may lead to shrouded neurodegeneration as part of the chronic process of postoperative neurocognitive decline (POCD) [9,14,15,16,17,18,19,20,21,22,23,24,25].

The progression of POCD determines the long-term cognitive outcomes of cardiac surgery [5,13,26,27]. POCD is characterized by an increase in the “classical” neurodegeneration markers tau, p181 tau, and amyloid β1-40 and β1-42, all linked to the emergence of Alzheimer’s disease (AD) and similar dementias [28,29,30,31]. These markers were studied extensively during the acute period of cardiac surgery; however, there are fewer data reflecting their persistence during recovery [23]. Interestingly, an increase in non-classical cognitive decline was observed in the peri-operative period [5,15,16,17,19,23,24,32,33]. Concomitantly, peri-operative delirium, vascular dementia, Parkinson’s-like disease, and other representations of central nervous system (CNS) dysfunction are increasingly recognized as cardiac surgery complications [9,16,17,18,23,24]. Delirium and atypical dementia may present a different clinical spectrum from classical AD-like dementia, yet their debilitating effect on long-term recovery is profound [13,32,34]. While the role of “classical” markers in POCD has been studied, the knowledge of the peri-operative dynamics of “non-classical” markers is lacking. The significant overlap in the presence of either type of markers (classical vs. non-classical) further complicates the understanding of POCD. Kallikrein-6 (KLK6) predicts the onset of neurocognitive decline in Alzheimer’s disease (AD), vascular dementia, and subarachnoid hemorrhages [35,36,37]. Neurogranin (NRGN) is related to synaptic injury, moderate cognitive decline, traumatic brain injury, and cerebral ischemia [29,38,39,40]. Chitinase 3-like 1 protein (YKL-40) is linked to astrocyte damage and signifies chronic vascular ischemia secondary to inefficient vascular remodeling [20,41,42]. Neural cell adhesion molecule 1 (NCAM-1) serum abnormalities were demonstrated in epilepsy and linked to Parkinson’s disease and autoimmune encephalitis [43,44,45,46,47]. While some of these non-classical markers were studied peri-operatively, long-term data are missing [30,48,49,50,51].

The progression of postoperative neurodegeneration depends on various processes, including the migration of peripheral leukocytes into brain parenchyma. This is driven by chemokine ligand 28 (CCL28) or monocyte chemoattractant protein-1 (MCP-1), especially if hypoxemia is present [16,34,51,52,53,54,55,56,57,58]. Perioperative damage of the blood–brain barrier and subsequent vasculitis and vascular remodeling further facilitate leukocyte infiltration into the CNS [59,60]. Peripherally driven inflammation via leukocytes activates microglia and astrocytes, boosting local neuroinflammation, as evidenced by the release of TDP-43, YKL-40, and tenascin [41,42,56,57,58,61,62,63,64,65,66,67]. This process is self-sustained even after an initial insult resolution, creating a risk of long-term neurodegeneration. Therefore, peri-operative insult, peripheral inflammation, and neuroinflammation synergize, leading to various brain injuries and several POCD subtypes [9,13,22,68]. This heterogeneity may be reflected in the composition and dynamic resolution of the classical and non-classical neurodegenerative markers.

The multiple pathways leading to POCD exacerbate the “classic-markers”-related symptoms of AD. Concomitantly, several non-classical markers of neurodegeneration may also contribute to POCD progression [15,23,24,25]. While the emergence of clinically apparent cognitive decline may be delayed for years, these same inflammatory processes also contribute to a higher risk of cerebrovascular accidents (CVA) in the peri-operative period [9,13,24,68]. In particular, the elevation of YKL40, fibroblast growth factor 2 (FGF2), and FGF23 is linked to blood vessel remodeling, as it is a factor of CVA emergence [48,69].

Several factors moderate the confluence of smoldering peripheral inflammation, local neuroinflammation, and vascular remodeling [42,51,70]. During surgery, neuroprotective mechanisms, such as fetuin and clusterin, trigger receptors expressed on myeloid cells 2 (TREM-2), ApoE, and the brain-derived neurotrophic factor (BDNF), dampening peripheral surgical inflammation and local neuroinflammatory stress [71,72,73,74,75,76,77,78,79]. However, their dynamics after cardiac surgery are mainly unknown despite their roles in various neurodegenerative processes [71,72,80,81]. It is possible that the prolonged depletion of these protective mechanisms may exacerbate neurological damage after cardiac surgery.

Herein, we investigated the serum milieu of neuroprotective factors (clusterin, fetuin, apoE, and BDNF) vs. neurodegenerative markers (NF-L, NF-H, τau, τaup181, amyloid β1-40, amyloid β1-42, YKL-40, TDP-43, NRGN, S100, and GFAP) in patients undergoing cardiac surgery. In order to relate the changes in these markers to the functional status of blood vessels, we studied vascular inflammatory (CCL28) and remodeling markers (FGF-2) [69]. The serum levels of Hsp-70 were used to assess the ongoing leak of DAMP, a potential stimulus for the immune system. We decided to apply cluster analysis to analyze the changes in the biomarkers holistically [9,18]. The longitudinal design of this study estimates marker dynamics over time and accounts for comorbidities [26,29,30]. We also assessed the incidence of CVA and cognitive dysfunction in patients. We hypothesized that persistent decreases in neuroprotective markers were linked to the persistence of peripheral inflammation (serum IL-6, IL-8, and CRP) and neuro-inflammation (TDP-43 and YKL-40).

## 2. Results

### 2.1. Assessment of Neurodegenerative Markers

**Figure 1:**
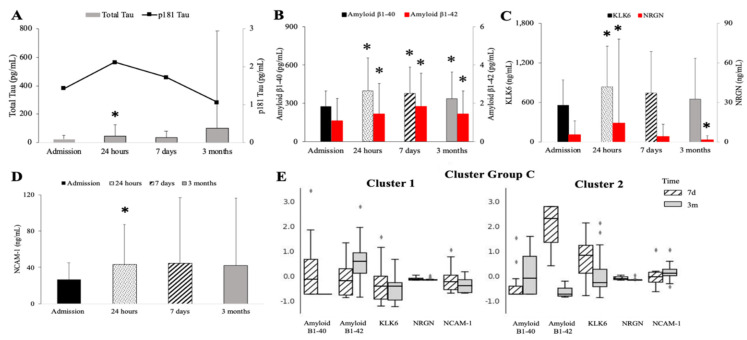
Time course of neurodegenerative markers after cardiac surgery. Levels of total τau (**A**, grey bars), p181tau (**A**, line), amyloid β1-40 (**B**, black bars), amyloid β1-42 (**B**, red bars), KLK6 (**C**, black bars), NRGN (**C**, red bars), and NCAM-1 (**D**) at baseline, 24 h, 7 days, and 3 months. (**E**) Cluster analyses of Cluster 1 (XX patients) and Cluster 2 (XX patients) with levels of amyloid β1-40 and β1-42, KLK6, NRGN, and NCAM-1 at 7 days (striped bar) and 3 months (solid bar). * *p* < 0.05. ◊ signifies values outside the nominal range or outlier.

A total of 158 patients were included in this study. An assessment of their neurodegenerative markers prior to surgery and at 24 h, seven days, and three months post-surgery is shown in Figure 1. The total level of τau was significantly elevated at 24 h but returned to baseline levels at seven days and three months post-surgery. The 3-month levels of total τau had high variability due to a few individuals with profoundly elevated levels (Figure 1A). The p181τau levels fluctuated at each follow-up but did not reach significance (Figure 1A). The Amyloid β1-40 and β1-42 serum levels were significantly increased at 24 h, seven days, and three months post-surgery when compared to the baseline levels (specific baseline level pg/mL) (Figure 1B). Serum S100 levels were highly variable, but they significantly peaked in a few patients 24 h after surgery (t_baseline_ = not detectable; t_+24hr_ = 1.6 ± 6.82 *; t_+7d_ = 0.2 + 3.79; t_+3m_ = not detectable; F[60;3] = 8.33; *p* = 0.0369). The dynamics of atypical neurodegeneration followed a somewhat uniform pattern. The levels of KLK-6, NRGN, and NCAM-1 significantly increased at 24 h before returning to baseline levels, although the NRGN levels dropped significantly below baseline levels at three months post-surgery (Figure 1C,D). The cluster analysis demonstrated two clusters differentiated by the predominance of amyloid β1-42 and amyloid β1-40, but lower KLK6 and NCAM-1 levels and a significant heterogeneity of NRGN (Figure 1E).

**Figure 2:**
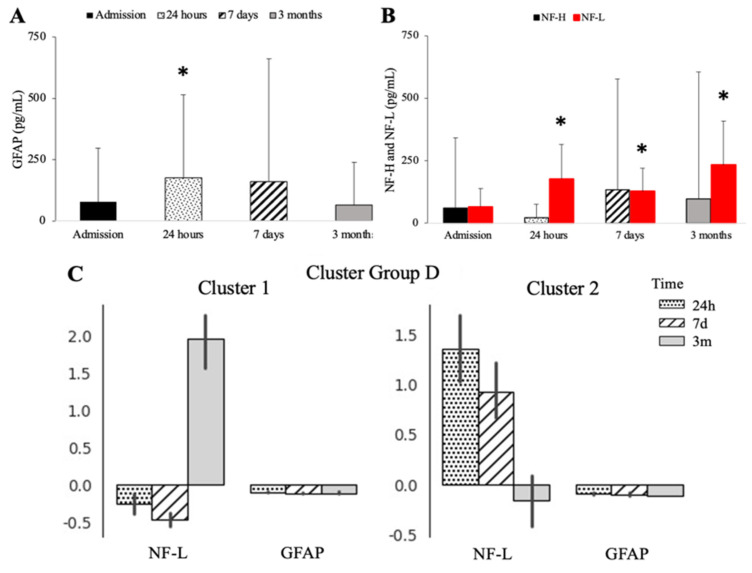
Post-operative changes in neuroinjury markers. Levels of GFAP (**A**), NF-H (**B**, grey bars), and NF-L (**B**, red bars) at baseline, 24 h, 7 days, and 3 months. (**C**) Cluster analyses separated by levels of NF-L and GFAP at 24 h (dots), 7 days (striped bar), and 3 months (solid bar). * *p* < 0.05.

**Figure 3:**
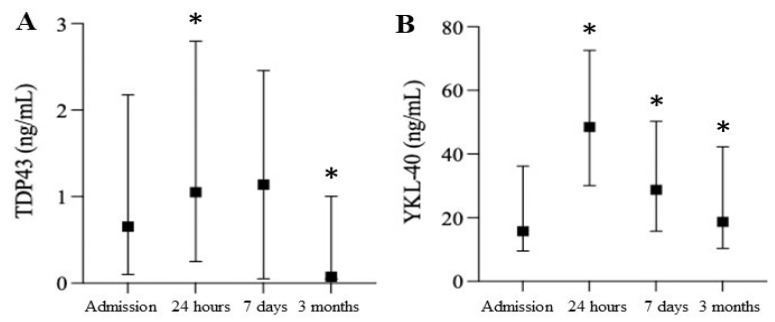
Time course of neuroinflammation markers after cardiac surgery. Levels of TDP43 (**A**) and YKL-40 (**B**) at baseline, 24 h, 7 days, and 3 months. * *p* < 0.05.

The levels of serum glial fibrillary acidic protein (GFAP) significantly increased 24 h post-surgery (Figure 2A). The NF-L levels were significantly elevated at all time points, while NF-H levels were highly variable without reaching significance (Figure 2B). The UC-HL levels demonstrated a delayed decrease at 7 days before returning to baseline (data not shown). Two clusters were identified based on the neuro-injury patterns: patients with persistently high serum levels of NF-L and GFAP and others with low and diminishing levels of GFAP (Figure 2C). Cluster 1 demonstrated a delayed increase in NFL at three months, while Cluster 2 initially had high NF-L levels that declined three months post-surgery. The GFAP levels were consistent at each follow-up. The levels of TDP-43 were significantly elevated during the peri-operative period at 24 h and then significantly decreased to below baseline levels at three months (Figure 3A). The serum YKL-40 levels were significantly elevated at all times after surgery (Figure 3B).

### 2.2. Severe Depletion of Neuro-Protective Markers Is Seen after Cardiac Surgery

**Figure 4:**
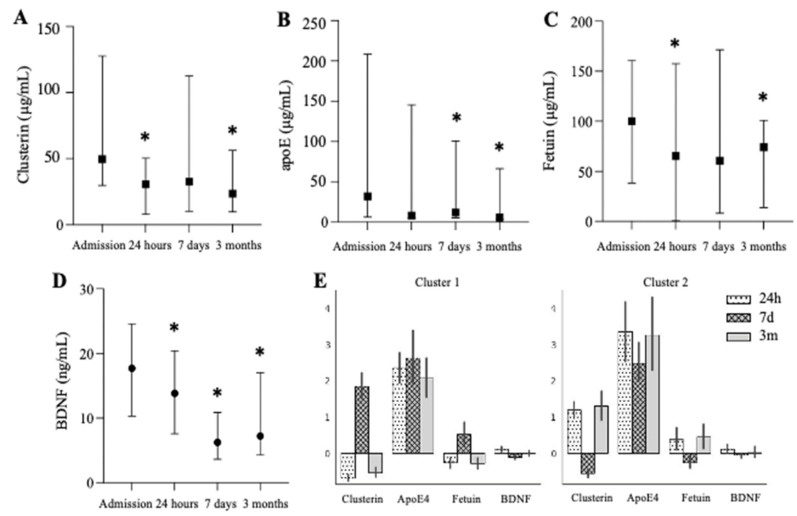
Temporal dynamics of neuroprotective markers after cardiac surgery. Levels of clusterin (**A**), apoE (**B**), fetuin (**C**), and BDNF (**D**) at baseline, 24 h, 7 days, and 3 months post-surgery. Two clusters of neuroprotective data were identified across studied patients (**E**). * denotes *p* < 0.05 as compared to admission value.

All markers of neuroprotection were significantly depressed, albeit with marker-specific dynamics. The clusterin levels significantly decreased at 24 h and three months compared to the baseline (Figure 4A). The ApoE levels were significantly decreased at seven days and three months compared to the baseline (Figure 4B). The ApoE changes in serum levels did not accompany these changes in the lipid profile, including LDL-c and HDL-c (data not shown). The fetuin levels were significantly decreased at 24 h and three months post-surgery, while the levels of BDNF were significantly diminished at all times (Figure 4C,D). A cluster analysis demonstrated the significant dynamics of clusterin and fetuin over time, while the ApoE levels remained constant in each cluster (Figure 4E).

### 2.3. Smoldering Vascular Inflammation Persists after Cardiac Surgery and Is Accompanied by CNS Leakage of the DAMPs

All the patients demonstrated a significant increase in serum IL-6 at 24 h (IL-6t_baseline_ = 35.6 ± 43.33; IL-6t_+24hr_ = 387.0 ± 326.49; IL-6t_+7d_ = 17.8 ± 16.87 *; IL-6t_+3m_ = 26.5 ± 43.48) and IL-8 at all time points (IL-8t_baseline_ = 12.9 ± 29.71; IL-8t_+24hr_ = 31.5 ± 49.76 *; IL-8t_+7d_ = 34.4 ± 79.71; IL-8t_+3m_ = 34.4 ± 79.71) immediately following cardiac surgery. There was a weak correlation between serum IL-6 and IL-8 levels (*r*^2^ = 0.26, CI95: 0.04–0.44; *p* = 0.0011).

**Figure 5:**
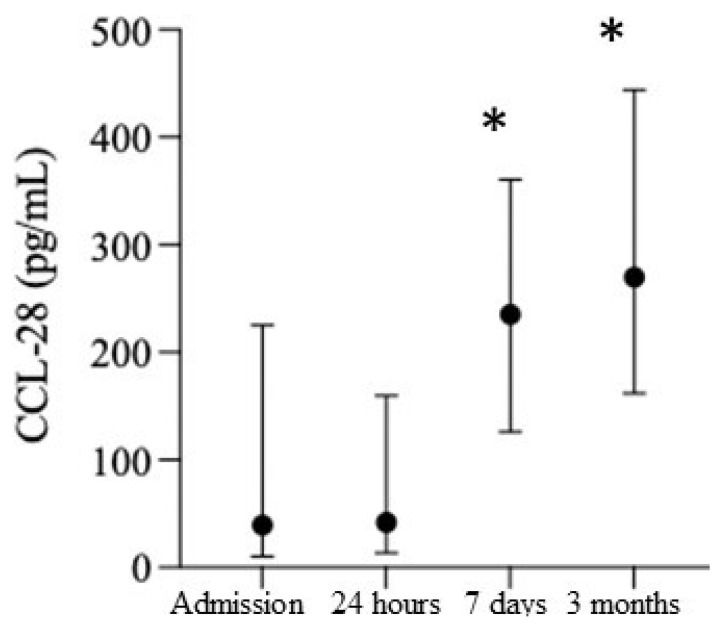
Time course of smoldering vascular inflammation marker after cardiac surgery. CCL-28 levels at baseline, 24 h, 7 days, and 3 months post-surgery.* *p* < 0.05.

The CRP levels significantly increased post-surgery at at all time points, (CRP_tbaseline_ = 139.1 ± 270.79; CRP_t+24hr_ = 858.9 ± 635.0; CRP_t+7d_ = 882.8 ± 562.72; CRP_t+3m_ = 369.2 ± 517.06; F[29;3] = 35.3; *p* = 0.00000). The CRP serum levels did not significantly correlate with the duration of anesthesia or surgery (data not shown). Similar to CRP, the CCL28 levels were also significantly increased at all time points (Figure 5). As an assessment for the leakage of danger-associated molecular patterns (DAMPs), HSP-70 showed significantly elevated levels at 7 days and 3 months post-surgery; however, there was high variability across all the timepoints (Hsp-70t_baseline_ = 0[0;0]; HSP-70t_+24hr_ = 0.0[0;0.21]; HSP-70t_+7d_ = 0.38[0;0.87] *; HSP-70t_+3m_ = 0.27[0;0.71] *; F[14;3] = 15.37; *p* = 0.00153).

### 2.4. Peri-Operative Management and Changes in Neuroinflammatory, Neuroprotective, and Neurodegenerative Markers

Peri-operative management, anesthesia, and surgical data (duration of anesthesia, duration of surgery, estimated blood loss, the volume of transfusion, and crystalloids given during anesthesia and within 24 h of ICU admission) did not correlate with classical or atypical neurodegeneration, neuro injury, neuroinflammation, peripheral nerve damage, or neuroprotective markers (data not shown). However, the length of stay in the hospital significantly correlated with τau_+24hr_ (*r*^2^ = 0.46; *p* = 0.0001), τau_+7d_ (*r*^2^ = 0.21; *p* = 0.046), τaup181–183_+24hr_ (*r*^2^ = 0.65; *p* = 0.0001), and UC-HL_+24hr_ (*r*^2^ = 0.51; *p* = 0.0001).

### 2.5. Incidence of CVA and Cognitive Dysfunction

Three patients expired by day 28 followed by an additional three patients at 3 months post-surgery, precluding any analysis. A total of 7.6% of patients had a history of stroke prior to surgery, with 6.0% experiencing a stroke within 48 h and 8.2% within one year after surgery. Neither demographic data nor pre-existing clinical conditions were significantly different between patients with an uneventful postoperative recovery and those suffering from stroke (Supplemental Table S1). Interestingly, the pre-surgery Charleston Comorbidity Index (CCI) scores were significantly lower in the patients with acute peri-operative stroke or a history of stroke (Supplemental Table S1). In addition, the levels of packed red blood cells and fresh frozen plasma significantly differed between the patients with new-onset CVA vs. those without complications (Supplemental Table S1).

There was a significant decrease in the Katz Index of Independence in Activities of Daily Living when comparing patients before and after surgery [82]. However, the results were highly non-parametric (data not shown). However, when the results were compared longitudinally, a small number of patients showed significant decreases when compared to the baseline (6 ± 0 vs. 5.7 ± 0.49; t[17] = 2.58; *p* = 0.02). The subjective perceptions of cognitive function, sleep, and memory problems were assessed as more favorable after surgery (Supplemental Figure S1).

## 3. Discussion

This is the first study demonstrating the long-term, post-surgical decline of neuroprotective markers (clusterin, ApoE, fetuin, and BDNF) with concomitant and longitudinal increases in neuro-injury (NF-L) and neurodegenerative markers (tau, NCAM-1, and amyloid β1-42) at the same time. In addition, the traits of smoldering vascular inflammation were present long-term, as evidenced by the CRP and CCL28 levels. We examined the outcomes three months post-surgery via direct follow-ups and an EMR review, an emerging trend intended to report more meaningful outcomes [16,83]. In addition, the study’s longitudinal design allowed for the moderation of inter-individual variability.

The studied neuroprotective markers work through various mechanisms during several types of peri-operative insults [9,17,18,23]. BDNF protects against ischemic injury [73]. Clusterin and ApoE limit amyloid retention in Alzheimer’s disease and mitigate cerebral microbleeds [71,77,78,81]. Fetuin is an anti-inflammatory molecule [75]. Both fetuin and clusterin reportedly dampen innate immunity by scavenging damage-associated molecular patterns (DAMPS) and moderating immunological activation [84,85,86,87]. Cluster analyses revealed that fetuin and clusterin are depleted in some patients alongside the persistent elevation of Hsp-70, providing stimulation for the immune system [88]. Though the source of Hsp-70 in our patients is unknown, the depletion of clusterin and fetuin exaggerated its stimulatory effects [75,76,77,78,81,84]. Consequently, these conditions favor neurodegeneration and neuroinflammation [16]. Additionally, our study suggests that some neuroprotective mechanisms may be chronically diminished after a stroke, rendering individuals prone to subsequent strokes. The etiology of the decrease in neuroprotective markers is unclear. The liver produces these markers, but the evidence of liver injury was not present in this study, according to the EMR review [71,84,85,86,87]. Lipid profile alterations have been frequently observed in acute illnesses, including surgery [89]. However, in our study, the depletion of ApoE occurred while the lipid profile was being restored. We did not measure the ApoE allotypes in our population, but an unintentional selection bias may have influenced our results [90,91]. Finally, we observed that peri-operative transfusion resulted in the depletion of some of the neuroprotective markers. This may account for abnormal peri-operative marker levels, but not long-term alterations in clusterin or fetuin.

Our data suggest that non-specific peri-operative inflammation is not fully resolved at three months, as demonstrated by the persistent level of CRP [16]. At the same time, peripheral leukocytes are activated via Hsp-70 [88,92] and an increase in CCL28, facilitating their entrance into the brain parenchyma, particularly three months after surgery. Alternatively, or synergistically, local microglia fail to de-activate for the long-term after cardiac surgery [14,19,61,62]. Cumulatively, all these processes fuel the pro-neuroinflammatory environment, as evidenced by the postoperative dynamics of YKL-40 and TDP-43 [30,42,48,49,65].

Ongoing inflammation and a composite decline in all three protective factors (clusterin, fetuin, and apoE4) create conditions favoring neuro-injury in our study. Subsequently, NF-L serum levels were consistently elevated, while GFAP demonstrated heterogeneity only in the cluster analysis. NF-L was previously reported as an important marker predicting postoperative cognitive impairment in a quasi-metanalysis enrolling several patient populations and types of surgery [32]. The surveillance periods in these studies were short, not exceeding seven days in most studies. The results of the analysis of the serum NF-L changes were related to the time on bypass or other mechanical burdens of surgery. However, our study suggests that a decline in neuroprotective markers, ongoing inflammation, and increased NF-L levels may be the underlying cause. In addition, the cluster analysis suggested that the neuro-injury process is heterogenous across the studied patients and may extend beyond the three months of follow-up.

We observed an increase in serum amyloid levels, as previously described, but our study cohort is significantly more extensive and accounts for several peri-operative variables [22,25,78,81]. Our prior study also showed changes in serum total tau, but a much more sensitive technique was used [19]. However, the increase in the amyloid, tau, and p181 tau levels in the peri-operative period suggests the release of the already accumulated markers secondary to neuron destruction during the peri-operative insult [9,24,25]. The subsequent persistence of the amyloid release only suggests that persistent neuroinflammation and unopposed peripheral inflammation may play a role [17]. Contrary to our initial hypothesis, we observed only transient increases in atypical neurodegenerative markers (KLK6, NRGN, and NCAM-1) [29,35,36,37,38,39,43,67,93]. Interestingly, these markers were linked to the emergence of postoperative delirium and were found to be present together with the tau protein [36,94,95,96]. Our study suggests that peri-operative neuronal injury may have distinctive markers and symptom profiles. However, the translation of the dynamic peri-operative profile of classical and non-classical markers into the emergence of cognitive decline needs to be established [33,35,38,41,67,93].

The lack of a correlation between surgical stress markers (time on bypass and length of surgery) and several neurodegenerative and neuro-injury markers suggests that cardiac surgery is a stimulant that triggers maximal activation. Others have recommended similar suggestions; thus, the overwhelming invasiveness of the procedure results in an overall immunological activation and subsequent neuronal damage [1,15,16,17,29,38,39,65,92]. Interestingly, surgery with fewer insults insult triggers a somewhat lower incidence of PCOD, delirium, and changes in neurodegenerative markers [5,17,18,51,70].

The clinical correlates of our study are of a pilot nature. Most peri-operative strokes reported in our study occurred early. This suggests that peri-operative management, pre-existing vulnerability, and the acute depletion of protective markers may play a synergistic role [26,27]. The transfusion volume correlated with the emergence of peri-operative stroke. Considering that the protective biomarker levels were most pronounced at three months, it can be inferred that blood loss and blood pressure fluctuation play a more significant role in peri-operative stroke [9,12,17,25]. On the other hand, a persistent decline in neuroprotective factors may increase the patients’ vulnerability to delayed CVA. However, the incidence of delayed CVA was not enough to conduct a comparison of the levels of neuroprotective markers and the incidence of CVA. Although we demonstrated some changes in the KATZ outcomes, the patients were assessed early after surgery. POCD should be assessed at least three months after surgery, optimally at 12 months [17]. Furthermore, the differences between the pre and post-scores were subtle and only significant if the data were compared longitudinally. We did not measure the incidence of peri-operative delirium, a common occurrence resulting in prolonged cognitive decline. However, we assessed the subjective perception of sleep quality, which diminished in some individuals. Similarly, self-reported cognitive decline was diminished in some patients. These subjective perceptions need verification through objective testing.

Our data are consistent with some prior observations. Amyloid elevations post-surgery were reported previously in a study enrolling 54 male patients undergoing on and off-pump revascularization [22]. Interestingly, patients subjected to the off-pump procedures experience a lower value change in amyloid levels than the presurgical levels. Similar data are available for τau and τaup181 even though different modalities were used to measure cytokines in several studies across different cardiac surgery types, suggesting that tau is quite commonly affected by the surgical conditions [22,32,78,81]. S100 was demonstrated to be elevated during the acute surgical period and highly dependent on pre-existing brain damage, but in our study, it fluctuated greatly [97]. Finally, we previously reported the time-dependent elevation of specific neurodegenerative markers (tau, GFAP, and UC-HL) using much more sensitive technology [14].

There are several limitations of our study. First, the patient cohort consisted primarily of male patients. Though we did not have sex-related differences, a more balanced representation could be more revealing. In this study, we were unable to gauge the duration, repetitiveness, and depth of the hypotension and hypoxic episodes [10,11,12,57,98]. Several markers are not exclusive to brain damage, such as YKL-40, which is elevated in atherosclerosis, cardiac illness, and diabetes [41,48]. Some of the markers utilized in this study are linked to the neurodegenerative process, but whether their roles are causative is unclear [16,28,29,36,42,72,86]. S100 is considered a neurodamage marker, but some of its features are also neuroprotective [97]. The neuroprotective markers were measured in serum; however, measuring them in cerebrospinal fluid might have been more relevant to neuroprotection. A significant portion of our patients had pre-existing diabetes and advanced atherosclerosis, potentially compounding the risk of CVA and POCD [16,66]. The patients were not screened for pre-existing dementia, and the level of cognitive decline was not quantified. We utilized ELISA to measure several markers, but SIMOA may be a more sensitive technique that could capture more accurate data. However, increasing the sensitivity of the technique would result in more accurate results rather than changes in the overall trends. Although we tested cognitive function, the daily activity index, sleep, and overall post-procedure satisfaction, more rigorous testing is needed. We did not account for the effects of the surgical operator and their experience translating into a decreased degree of trauma and shorter surgery. Most importantly, anesthesia significantly affects the release of neurodegenerative markers. These effects are often contradictory depending on the agents and neurodegenerative markers [99,100,101]. It is challenging to interpret these studies unequivocally since the effect of anesthetic agents must be separated from overall cerebral perfusion and flow [102,103]. In addition, several studies observe the effect in animal models, with a potential difficulty in relating the findings to humans [23,51,53,58,73,99,101,103].

The strength of this study is the large cohort of patients, creating a longitudinal data set. The operating group of surgeons was small, but they have significant, long-term experience in a large-volume surgical center. The patient selection was diverse with respect to surgery types. The institution’s pre-, intra-, and postoperative care was highly standardized. Robust technologies were used to measure the biomarkers.

## 4. Materials and Methods

### 4.1. Consent

University of Pennsylvania Institutional Review Board approved the study (#815686). All patients scheduled for non-emergent cardiac surgery were approached for consent.

### 4.2. Patient Population

A total of 158 patients met the inclusion criteria of undergoing non-emergent cardiac surgery, were aged ≥ 18 years, and provided informed consent. The exclusion criteria included age < 18 years, emergent surgery, pre-existing immunosuppression, or lack of consent. Patient characteristics are summarized in Table 1. Totals of 87, 78, and 69 patients provided follow-up samples at 24 h, 7 days, and 3 months post-surgery, respectively.

### 4.3. Clinical Data Collection

Demographic and clinical data were obtained from electronic health records (EHR), including surgical, anesthesic, and peri-operative records. Morphine equivalents were calculated for opioids given in the first 24 h following surgery. APACHE II score was calculated upon admission to the ICU and 24 h later [104,105]. The diagnoses of cerebrovascular events pre- and peri-operatively were extracted from medical records by manual chart review. Mortality was defined at 28 days.

### 4.4. Study Procedure

After consent was secured, patients’ blood was collected before the onset of surgery (t_0_). Subsequent blood procurements took place 24 h (t_24hr_) and seven days (t_7d_) post-surgery, with a final follow-up at three months (t_3m_).

Blood was collected from arterial lines during the hospital stay, from the venous system using central lines, or was manually drawn using the Vacutainer™ system (BD; Franklin Lakes, NJ, USA). Blood was collected and stored at 4 °C until further processing within 2–4 h. Serum was isolated by centrifugation for 10 min, at 4 °C, at 1200× *g,* and was aliquoted. Samples were stored at −80 °C until further processing.

### 4.5. Assessment of Biomarkers

Biomarkers (τau, τau p181–183, amyloid β1-40 and β1-42, S100, KLK6, YKL-40, NRGN, NF-H, UC-HL, GFAP, YKL-43, TDP-43, NCAM-1, apoE4, BDNF, fetuin, clusterin, RANTES, FGF2, CRP, Hsp-70, and IL-6) were measured using the multiplex technique per the manufacturer’s recommendation (Thermofisher, Waltham, MA, USA). The data were collected using 3DFlexAmp (Luminex, Toronto, ON, Canada). Serum NF-L was measured using ELISA (American Research Products, Waltham, MA, USA) with 100 microliters of the isolated serum run for each sample and optimized for the standard curve. For those run using the multiplex technique, 25 microliters of isolated serum was run for each sample and optimized for the standard curve.

### 4.6. Cognitive and Daily-Living Performance Testing

Patients were given the KATZ Index of Independence in Activities of Daily Living (ADL) and daily living assessment questionnaires at the time of consent and three months after surgery [82]. In addition, patients were asked to ascertain their frequency of sleep disturbances, perception of memory, and quality of life at 3 months compared to before surgery (−1: worse compared to before surgery; 0: the same as before surgery; +1: better than before surgery).

### 4.7. Statistical Analysis

Shapiro–Wilk and K–S tests were used to test the normality and assess the distribution of variables. Parametric variables are expressed as mean ± SD and compared using *t*-Student for two variables, while ANOVA was used for multiple comparisons. For non-parametric variables, median (M_e_) and interquartile ranges (IR) were utilized, with U-Mann–Whitney statistics employed to compare such variables. The data were analyzed as dependent, paired samples with Bonefforri’s correction for multiple comparisons. *k*-means with pairwise elimination of variables were utilized for clustering (R-package). *r*^2^ and ρ Spearman correlation coefficients were used to assess relationships for parametric and non-parametric variables, respectively. Both-sided *p*-values less than 0.05 were considered statistically significant for all tests unless a specific null hypothesis was formulated. Statistical analyses were performed with Statistica 11.0 (StatSoft Inc., Tulsa, OK, USA) or Statistical Package for the Social Sciences v26 (IBM, Amon, NY, USA).

## 5. Conclusions

Our study demonstrated the imbalance between several neurodegenerative, neuroinjury, and neuroprotective markers up to three months after elective cardiac surgery. Our study suggests that peri-operative injury and inflammation, if left unchecked or unresolved, may become a chronic process leading to an increased risk of cognitive decline.

## Figures and Tables

**Table 1:** Patient demographics and clinical characteristics.

Demographics		*N* = 158
**Age (years)**		
Mean ± SD		64.2 ± 12.1
	Over 60 [%]	70.2%
Sex		
	Male [%]	74.05%
	Female [%]	25.31%
	Not reported [%]	0.64%
Race		
	Black [%]	3.8%
	White [%]	87.0%
	Other/Asian/Unknown [%]	9.2%
**Pre-Existing Conditions**		
Weight (kg) [mean ± SD]		86.1 ± 21.71
BMI (mean ± SD)		28.4 ± 6.09
Charleston Comorbidity Index [mean ± SD]		3.89 ± 2.13
ACS/MI [%]		13.3%
CHF [%]		19.6%
PVD [%]		9.4%
CVA/TIA [%]		7.6%
Dementia [%]		0%
COPD [%]		6.96%
DM [%]		27.8%
**Anesthesia and Surgery Data**		
Duration of anesthesia (min) [mean ± SD]		374.8 ± 107.77
Duration of surgery (min) [mean ± SD]		265.5 ± 100.74
Duration of cardiopulmonary bypass (min) [mean ± SD]		130.6 ± 65.69
Coronary artery bypass surgery [*n*]		108
Mitral valvuloplasty and replacement [*n*]		36
Aortic valvuloplasty and replacement [*n*]		60
Aortic aneurysm repair [*n*]		19
Other [*n*]		9
Estimated Blood Loss (mL) [mean ± SD]		205.5 ± 291
**Peri-operative management**		
**Transfusions during surgery**		
Packed red blood cells (mL) [mean ± SD]		120 ± 270
Fresh frozen plasma (mL) [mean ± SD]		87 ± 260
Total crystalloid during surgery (mL) [mean ± SD]		1297 ± 291
**Clinical Care during 24 h post-surgery**		
Packed Red Blood Cells (mL) [mean ± SD]		17 ± 85
Fresh Frozen Plasma (mL) [mean ± SD]		5.8 ± 75
Opioids Administration (mg) [mean ± SD]		698 ± 233
Benzodiazepine administration (mg) [mean ± SD]		3.67 ± 1.73
**APACHE II scores measured in ICU**		mean ± SD
1 h		16.8 ± 6.02
24 h		9.4 ± 4.91
48 h		9.1 ± 4.66
**Outcome at 28 days**		
LOS ICU (day) [mean ± SD]		4.36 ± 16.26
LOS Hospital (day) [mean ± SD]		10.3 ± 18.49
Discharged		87.3%
In the healthcare facility		6.39%
Mortality		1.26%

ACS = acute coronary syndrome, APACHE = Acute Physiology and Chronic Health Evaluation, BMI = body mass index, CHF = congestive heart failure, COPD = chronic obstructive pulmonary disease, CVA = cerebrovascular accident, DM = diabetes melitis, ICU = intensive care unit, IQ = interquartile range, LOS = length of stay, MI = myocardial infarction, PVD = peripheral vascular disease, SD = standard deviation, and TIA = transient ischemic attack.

## Data Availability

The datasets used and/or analyzed during the current study are available from the corresponding authors on reasonable request after IRB’s approval.

## Supplementary Materials

The following supporting information can be downloaded at: https://www.mdpi.com/article/10.3390/biomedicines10102364/s1, Table S1. Comparison of patients with pre-existing stroke, peri-operative stroke, or without any history of CVA. Figure S1. Cognitive assessments pre- and post-surgery. Distribution of cognitive function (A), sleep (B), and memory (C) assessment results. Pre-surgery baseline (bin center 0) compared with better (+1) or worse (−1) post-surgical assessment results.
