# Supplementary material for: Persistent Depletion of Neuroprotective Factors Accompanies Neuroinflammatory, Neurodegenerative, and Vascular Remodeling Spectra in Serum Three Months after Non-Emergent Cardiac Surgery
Source: Biomedicines. 2022 Sep 22;10(10):2364. doi: 10.3390/biomedicines10102364 (PMC9598177; doi:10.3390/biomedicines10102364)
Supplement: Supplementary file 1 [file biomedicines-10-02364-s001.zip › biomedicines-1811959-supplementary.pdf]

Table S1. Comparison of patients with pre-existing stroke, peri-operative stroke, or without any history of CVA.

	History of CVA (*n* = 12)	CVA After Surgery (*n* = 16)	None (*n* = 137)
**Demographics**			
**Age (years)**			
mean ± SD	58.9 ± 14.7	69.7 ± 7.8	70.3 ± 7.6
Over 60 [%]	75	92.3	100
**Sex**			
Male [%]	75	76.9	100
Female [%]	25	23.1	0
Not Reported [%]	0	0	0
**Race**			
Black [%]	8.3	7.7	25
White [%]	83.3	92.3	75
Other/Asian/Unknown [%]	8.3	0	0
**Pre-Existing Conditions**			
Weight (kg) [mean ± SD]	87.1 ± 23.0	86.0 ± 28.4	83.0 ± 15.9
BMI [mean ± SD]	28.9 ± 5.8	27.6 ± 7.3	26.8 ± 4.0
CCI [mean ± SD]	**4.5 ± 2.1 ***	**4.6 ± 1.6 ***	6.5 ± 1.3
ACS/MI [%]	16.7	23	50
CHF [%]	8.3	23	25
NT-BNP [mean ± SD]	5994.3 ± 8504.2	4651.8 ± 5861.7	3105.2 ± 4567.0
PVD [%]	8.3	15.4	25
COPD [%]	8.3	15.4	25
DM [%]	33.3	38.5	25
Hb1AC [mean ± SD]	5.8 ± 0.46	6.7 ± 2.2	6.1 ± 1.1
**Anesthesia and Surgery Data**			
Duration of anesthesia (min) [mean ± SD]	377.8 ± 114.4	418.0 ± 116.2	370.4 ± 104.8
Duration of surgery (min) [mean ± SD]	269.2 ± 99.5	305.4 ± 121.9	261.6 ± 98.3
Duration of cardiopulmonary bypass (min) [mean ± SD]	127.7 ± 56.7	167.4 ± 111.4	127.6 ± 61.5
Coronary artery bypass surgery (*n*)	12	9	129
Mitral valvuloplasty and replacement (*n*)	4	4	29
Aortic valvuloplasty and replacement (*n*)	3	2	56
Aortic aneurysm repair (*n*)	1	0	18
Others (*n*)	1	4	5
Estimated Blood Loss (mL) [mean ± SD]	262.5 ± 389.7	137.5 ± 43.3	205.3 ± 292.1
**Peri-operative Management**			
**Transfusions during surgery**			
Packed Red Blood Cells (mL) [mean ± SD]	9.0 ± 63.3	184.6 ± 357.9	100.7 ± 241.1
Fresh Frozen Plasma, (mL) [mean ± SD]	104.2 ± 225.1	269.2 ± 554.0	70.9 ± 215.9
Total crystalloid during surgery (mL) [mean ± SD]	1600.0 ± 797.5	1700.0 ± 892.6	1256.0 ± 528.8
**Clinical Care during 24 h post-surgery**			
Packed Red Blood Cells (mL) [mean ± SD]	50.0 ± 173.2	**115.4 ± 195.1 ***	9.0 ± 63.3
Fresh Frozen Plasma (mL) [mean ± SD]	0.0 ± 0.0	**69.2 ± 249.6 ***	0.0 ± 0.0
Opioids Administration (mg) [mean ± SD]	757.3 ± 270.1	660.7 ± 233.4	702.2 ± 233.4

Benzodiazepine administration (mg) [mean ± SD]	2.6 ± 6.8	2.5 ± 6.5	0.13	± 0.47

ACS = acute coronary syndrome , BMI = body mass index, CCI = Charlson Comorbity Index , CHF = congrstive heart failure, COPD = chronic obstructive pulmonary disease, CVA = , DM = diabetes melitis, MI = myocardial infarction, NT-BNP = N-terminal pro b-type natriuretic peptide, PVD = peripheral vascular disease, SD = standard deviation, * $p < 0.05$.

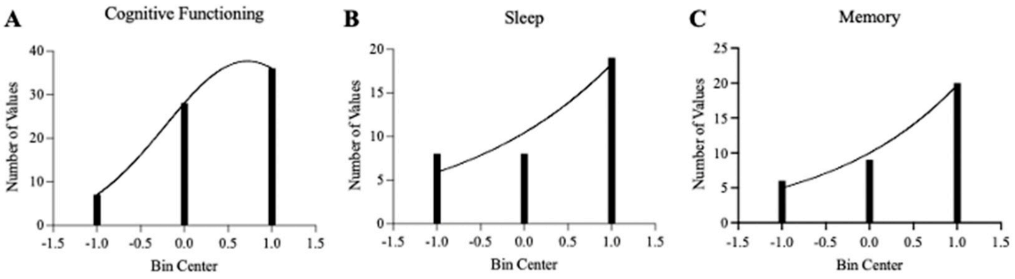

Figure S1. Cognitive assessments pre- and post-surgery. Distribution of cognitive function (**A**), sleep (**B**), and memory (**C**) assessment results. Pre-surgery baseline (bin center 0) compared with better (+1) or worse (−1) post-surgical assessment results.
